# Repeatability of Pentacam-derived intraocular lens decentration measurements and the level of agreement with OPD-Scan III: A prospective observational case series

**DOI:** 10.1371/journal.pone.0299064

**Published:** 2024-03-22

**Authors:** Xiaobao Liu, Wenjie Wu, Yulong Huang, Yabo Fu, Yue Huang, Qiong Li

**Affiliations:** 1 Shengli Clinical Medical College of Fujian Medical University; Fujian Medical University, Fuzhou, China; 2 Department of Ophthalmology, Fujian Provincial Hospital, Fuzhou, China; 3 Department of Ophthalmology, Longyan People Hospital of Fujian, Longyan, China; Democritus University of Thrace, GREECE

## Abstract

**Purpose:**

This study aimed to assess the repeatability of intraocular lens (IOL) decentration measurements obtained through Pentacam, based on corneal topographic axis (CTA) and pupillary axis (PA), and to evaluate the level of agreement between Pentacam and OPD-Scan III devices in measuring IOL decentration.

**Methods:**

In this prospective observational case series, three measurements were performed with Pentacam to evaluate the repeatability of the measurements. The analysis included the calculation of the mean and standard deviations (SD), conducting a repeated measures analysis of variance (rANOVA), and determining an intraclass correlation coefficient (ICC) to assess the repeatability of the measurements. Moreover, Bland-Altman analysis was employed to assess the agreement between Pentacam and OPD-Scan III devices in measuring IOL decentration. IOL decentration measurements were obtained with respect to both CTA and PA.

**Results:**

A total of 40 eyes from 40 patients were analyzed. The rANOVA revealed no significant difference among three consecutive measurements of IOL decentration obtained with Pentacam. The mean SD of all parameters ranged from 0.04 mm to 0.07 mm. With CTA as the reference axis, the ICC values for Pentacam measurements of IOL decentration were 0.82 mm for the X-axis, 0.76 mm for the Y-axis, and 0.82 mm for spatial distance. When using PA as the reference axis, the corresponding ICC values were 0.87, 0.89, and 0.77, respectively. The 95% limits of agreement for all IOL decentration measurements were wide when comparing Pentacam and OPD-Scan III.

**Conclusions:**

Pentacam demonstrated high repeatability in measuring IOL decentration with respect to both CTA and PA. However, due to poor agreement between Pentacam and OPD-Scan III measurements, caution should be exercised when using data interchangeably between the two devices.

## Introduction

The advancements in intraocular lens (IOL) design and prolonged lifespan of pseudophakic patients have led to greater expectations for positive visual and refractive outcomes following cataract surgery [1–3]. However, the decentration of IOLs can considerably impair postoperative optical quality and overall quality of life, particularly in cases involving intricate IOLs implantation, such as multifocal IOLs and toric IOLs. [4–6]. Therefore, precise measurement of IOL decentration is essential for estimation of postoperative visual performance after refractive cataract surgery.

Several techniques are currently employed for the measurement of IOL decentration [4, 5, 7–9]. One of these is the Scheimpflug method, which has garnered considerable prominence, featuring in nearly 30% of published studies focused on IOL decentration measurements [4, 10–12]. The Pentacam, a non-invasive device based on the Scheimpflug principle, can produce precise, high-resolution images that enable an accurate assessment of the IOL’s position. A newer device that has gained widespread use in recent years is the OPD-Scan III, which can detect IOL decentration and provide supplementary optical quality data in a single scan. This feature streamlines the measurement process and improves the reliability and accessibility of subsequent optical quality analysis [5, 7, 13–15]. Accordingly, the OPD-Scan III has become increasingly popular in the realm of IOL decentration measurements and optical quality analysis.

Historically, studies investigating the measurement of IOL decentration have predominantly relied on the pupillary axis (PA) as the reference axis, primarily due to its clinical ease of visualization [10, 16–18]. Regrettably, the inherent variability of pupil size and location undermines the precision and consistency attainable with this reference axis [19, 20]. Consequently, the corneal topographic axis (CTA), positioned close to the subject-fixated coaxially sighted corneal light reflex axis, has emerged as a proposed universal standard axis for decentration measurement [17, 21]. However, to date, no studies have elucidated the repeatability of IOL decentration measurements specifically focused on the CTA utilizing Pentacam. Furthermore, the interchangeability of Pentacam-based IOL measurements referenced to both CTA and the PA with those obtained from OPD-Scan III remains unknown.

Therefore, the primary objective of the present study was to assess the repeatability of IOL decentration measurements derived from the Pentacam, focusing on both the PA and the CTA. Additionally, a secondary aim involved evaluating the level of agreement among the Pentacam and the OPD-Scan III with regards to IOL decentration measurements.

## Materials and methods

**Fig 1:**
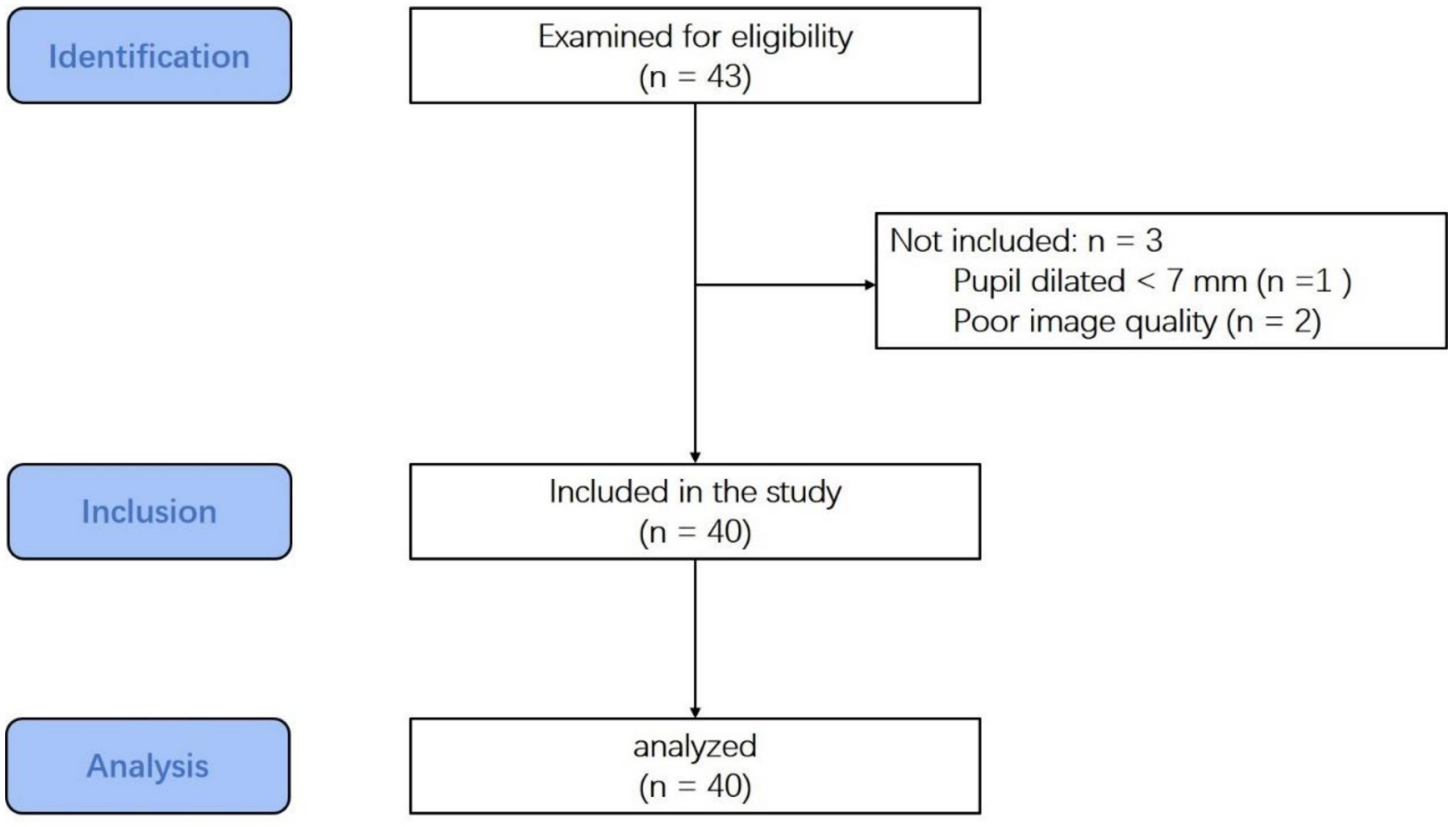
The study STROBE flowchart.

A STROBE flowchart is shown in Fig 1.

### Participants

This was a prospective observational case series, in which 43 eyes of 43 patients with age-related cataract were recruited from the Department of Ophthalmology, Fujian Provincial Hospital (Fujian, China), between October 1^st^, 2021 and January 1^st^, 2022. Patients with a history of trauma, uveitis, glaucoma, pseudoexfoliation syndrome, strabismus, dilated pupil diameter of less than 5.0 mm, axial length of less than 21 mm or more than 25 mm, keratoconus or other corneal disease, or previous ocular surgery or laser treatment were excluded. The study protocol was approved by the Institutional Review Board of Fujian Provincial Hospital on April 29^th^, 2020 (K2020-04-048), and all procedures were conducted in accordance with the principles outlined in the Declaration of Helsinki. Written informed consent was obtained from each patient prior to his or her participation.

All subjects underwent routine phacoemulsification and IOL implantation, with surgical parameters detailed in Table 1. At the 3-month follow-up visit, a comprehensive ophthalmic examination was conducted for each patient, including assessments of uncorrected visual acuity (UCVA), best corrected visual acuity (BCVA), slit-lamp microscopy, noncontact tonometry, fundus examination, and measurement of IOL decentration using both Pentacam AXL (Oculus, Wetzlar, Germany) and OPD-Scan III (Nidek Co., Ltd., Gamagori, Japan) devices.

**Table 1:** Characteristics.

Parameter	
Age (years)	68.97±6.57
Gender (male/female)	14/26
Sidedness (right/left)	17/23
Preoperative CDVA (logMAR)	0.86±0.53
Axial length (mm)	23.54±0.80
IOP (mmHg)	15.75±2.40
K1 (D)	43.79±1.43
K2 (D)	44.56±1.44
Km (D)	44.17±1.43
Specification of IOL	
Tecnis ZCB00	5
Tecnis ZMB00	16
Tecnis ZXR00	17
Proming A1-UV	2
IOL power (D)	22.00±2.11
Design	1-piece
Material	Acrylic
Optic diameter (mm)	6.00
Haptic	Modified C

CDVA, corrected distance visual acuity; IOP, intraocular pressure; K, keratometry; Km, mean keratometry; D, diopter; IOL, intraocular lens.

### Surgical technique

All cataract surgeries were conducted by a single surgeon (WJW) according to a standard procedure. A 2.4-mm temporal clear corneal incision and a side port incision were created. Subsequently, nuclear removal and cortical aspiration were performed within a precisely centered 5.5-mm anterior continuous curvilinear capsulorrhexis. Following the cataract removal and capsular polishing, IOL implantation was carried out. The IOL was carefully rotated to achieve a vertical position. Lastly, any remaining ophthalmic viscosurgical device was aspirated, and the incisions were hydrated.

### IOL decentration measurement

The measurement of IOL decentration was conducted using the Pentacam and OPD-Scan III devices following complete mydriasis achieved with a mixture of 1% tropicamide and 2.5% phenylephrine hydrochloride. Before the study, both devices were calibrated by their respective technical support services. The order of the measurements for the patients was determined using a computerized table of random numbers. All measurements were performed in a standard dim-light room. Two optometrists, who were blinded to the study protocol, were involved in the measurements: one for Pentacam imaging and the other for OPD-Scan III acquisitions.

To measure the IOL decentration using Pentacam, horizontal (0–180°) and vertical (90–270°) cross-sections of Scheimpflug images were captured three times consecutively. Before each measurement, careful attention was paid to head positioning and eye alignment. Patients were instructed to blink immediately before the measurements and were asked to briefly leave the headrest and chinrest between two consecutive measurements. An image was acquired only when the data quality statement indicated "OK" and the edge of the IOL was clearly visible. Subsequently, Image J software (Image J version 1.52, NIH, USA) was utilized for image processing.

The identification of the CTA and PA followed the method described by previous studies [10, 16]. Two arcs were adjusted to fit the anterior and posterior surfaces of the IOL, and the IOL center was determined as the midpoint of the IOL plane passing through the two intersections of the arcs. The position of the CTA was obtained by connecting the fixation point to the corneal vertex. The PA was defined as the line perpendicular to the cornea and passing through the midpoint of the two visible segments of the pupil.

**Fig 2:**
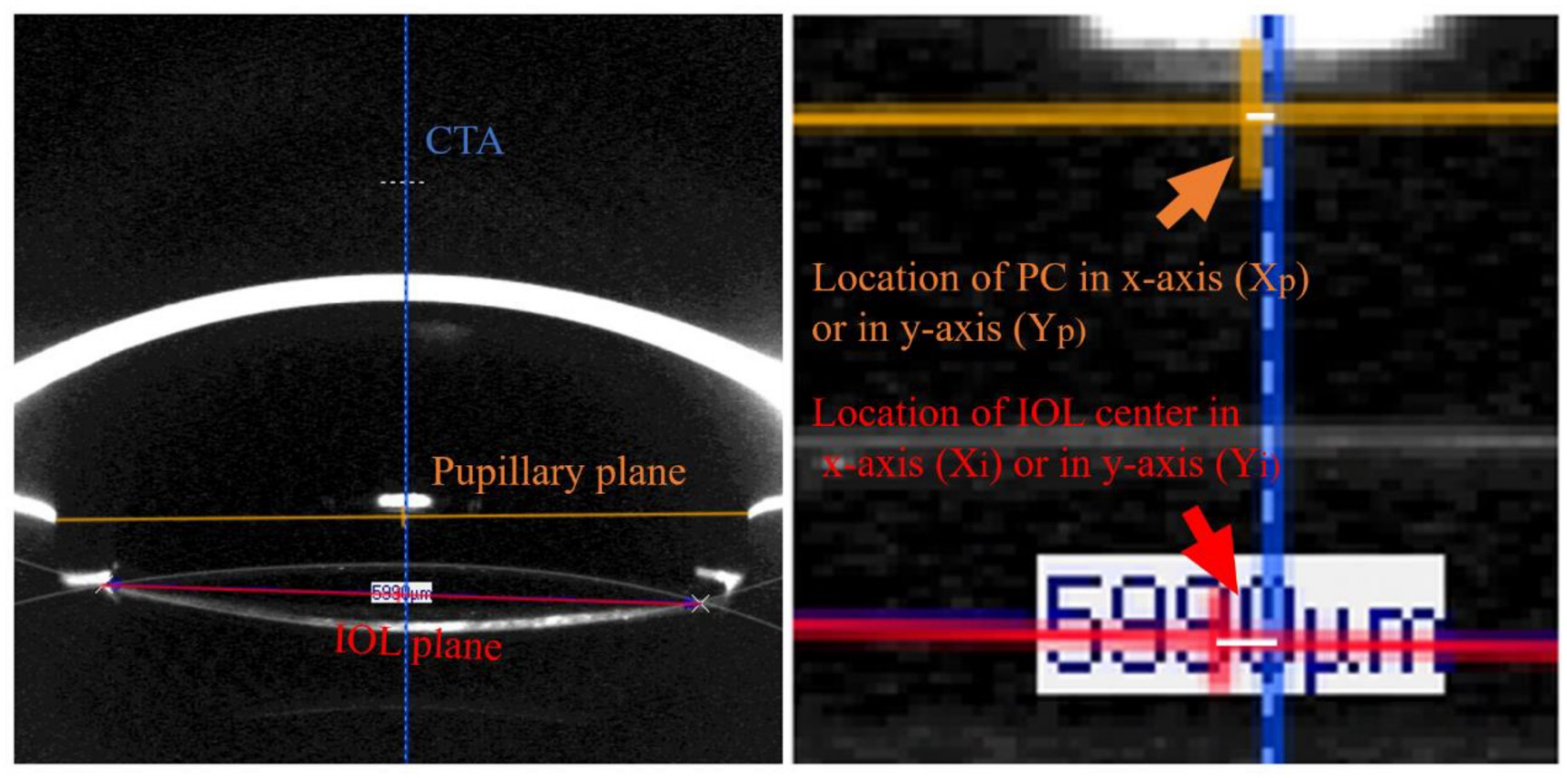
Definition for all parameters in Sheimpflug image using Pentacam. CTA, corneal topographic axis; IOL, intraocular lens; PC, pupil center. The blue line represents CTA. The orange line represents pupillary plane. The red line represents IOL plane.

Given the high agreement between Pentacam and OPD-Scan III in measuring corneal curvature, the corneal vertex was chosen as the origin of the coordinate system to determine the positions of the IOL center and pupil center in both devices [22]. Using a scale of 0.02 mm/pixel, the locations of the PC and IOL center were represented as (X_p_, Y_p_) and (X_i_, Y_i_), respectively (Fig 2). The parameters of IOL decentration with reference to CTA and PA were calculated as shown in Table 2. Positive horizontal coordinates represented nasal decentrations in the right eye and temporal decentrations in the left eye, while positive vertical coordinates indicated superior decentrations and negative values indicated inferior decentrations.

**Table 2:** Parameters definition and equation of IOL decentration measurement with Pentacam.

Abbreviated names	Full names	The equation
D_CX_	IOL decentration with reference of CTA in the x-axis	D_CX_ = X_i_
D_CY_	IOL decentration with reference of CTA in the y-axis	D_CY_ = Y_i_
D_C_	IOL decentration with reference of CTA in spatial distance	D_C_ = (X_i_^2^+ Y_i_^2^)^1/2^
D_PX_	IOL decentration with reference of PC in the x-axis	D_PX_ = (Xi—X_p_)
D_PY_	IOL decentration with reference of PC in the y-axis	D_PY_ = (Yi—Y_p_)
D_P_	IOL decentration with reference of PC in spatial distance	D_P_ = ((Xi—X_p_)^2^ + (Yi—Y_p_)^2^)^1/2^

IOL, intraocular lens; CTA, corneal topographic axis; PC, pupil center; X_i_, the distance between IOL center and corneal vertex in x-axis; Y_i_, the distance between IOL center and corneal vertex in y-axis; X_p_, the distance between PC and corneal vertex in x-axis; Y_p_, the distance between PC and corneal vertex in y-axis.

For OPD-Scan III, the locations of the IOL center and pupil center relative to CTA were directly obtained from the instrument as distances (d_i_’ and d_p_’, respectively) and orientations (α_i_ and α_p_, respectively) (S1 Fig). The parameters were calculated according to Table 3.

**Table 3:** Parameters definition and equation of IOL decentration measurement with OPD-Scan III.

Abbreviated names	Full names	The equation
D_CX_’	IOL decentration with reference of CTA in the x-axis	D_CX_’ = Sinα_i_ * d_i_’
D_CY_’	IOL decentration with reference of CTA in the y-axis	D_CY_’ = Cosα_i_ * d_i_’
D_C_’	IOL decentration with reference of CTA in spatial distance	D_C_’ = d_i_’
D_PX_’	IOL decentration with reference of PC in the x-axis	D_PX_’ = Sinα_i_ * d_i_’—Sinα_p_ * d_p_’
D_PY_’	IOL decentration with reference of PC in the y-axis	D_PY_’ = Cosα_i_ * d_i_’—Cosα_p_ * d_p_’
D_P_’	IOL decentration with reference of PC in spatial distance	D_P_’ = ((Sinα_i_ * d_i_’—Sinα_p_ * d_p_’)^2^ + (Cosα_i_ * d_i_’—Cosα_p_ * d_p_’)^2^)^1/2^

IOL, intraocular lens; CTA, corneal topographic axis; PC, pupil center; d_i_’, the spatial distance between IOL center and corneal vertex; α_i_, the orientation of IOL center based on corneal vertex; d_p_’, the spatial distance between IOL center and PC; α_p_, the orientation of IOL center based on PC.

### Sample size

The determination of the sample size was carried out with a significance level (α) of 0.01 and a power of 90%. Drawing from prior studies and our own research data, the average IOL decentration level observed was approximately 0.3 mm, which was within the acceptable range [4, 5, 7]. Based on previous research, clinically significant IOL decentration has been defined as decentration exceeding 0.4 mm [23, 24]. As a result, a clinically relevant mean difference of 0.1 mm was considered for the calculation. Based on the results obtained from a pilot study, which indicated a mean measurement error of 0.15 mm for IOL decentration measurement, the minimum required sample size was determined to be 34.

### Statistical analysis

Statistical analyses were conducted using SPSS for Windows Version 15.0 software (SPSS Inc., Chicago, IL, USA). All measurements were reported as mean ± standard deviation (SD). The statistical significance of differences between the three consecutive measurements obtained with the Pentacam was assessed using repeated-measures analysis of variance (rANOVA). A p-value less than 0.05 was considered statistically significant. Furthermore, the mean SD and intraclass correlation coefficients (ICCs) were calculated to evaluate the repeatability of the Pentacam measurements. A higher ICC value indicates better measurement consistency.

To assess the agreement between the measurements obtained with the Pentacam and OPD-Scan III, Bland-Altman plots were employed. The 95% limits of agreement (LoA) were determined as the mean ± 1.95 times the SD of the measurement difference between the two devices. In the Bland-Altman plots, the y-axis represented the difference between each pair of measurements, while the x-axis depicted the mean values. The central line on the plot represented the mean of the measurement differences between the two devices, and the broken lines indicated the 95% LoA.

## Results

In this study, three eyes were excluded due to a failure to achieve pupil dilation in one patient and poor image quality in two patients. Therefore, a total of 40 eyes from 40 patients were included in the analysis. The demographic data and surgical parameters of the patients are presented in Table 1.

At the 3-month postoperative follow-up, the mean IOL decentration measurements, with reference to the CTA using the Pentacam, were as follows: -0.17 ± 0.12 mm in the x-axis, 0.07 ± 0.11 mm in the y-axis, and 0.24 ± 0.10 mm in spatial distance. When using the PC as the reference, the mean IOL decentration measurements were -0.20 ± 0.14 mm, 0.09 ± 0.18 mm, and 0.30 ± 0.13 mm, respectively. A rANOVA indicated no statistically significant differences among the three consecutive measurements for all parameters obtained with the Pentacam. The measurements of D_CX_, D_CY_, D_C_, D_PX_, D_PY_, and D_P_ showed high repeatability, as indicated by ICCs ranging from 0.76 to 0.89, and mean SD ranging from 0.04 to 0.06 mm. For the OPD-Scan III, the IOL decentration measurements, with reference to the CTA, were -0.20 ± 0.22 mm in the x-axis, 0.28 ± 0.17 mm in the y-axis, and 0.25 ± 0.14 mm in spatial distance. When using the PC as the reference, the measurements were -0.32 ± 0.40 mm, 0.20 ± 0.35 mm, and 0.45 ± 0.27 mm, respectively (Table 4).

**Table 4:** Repeatability of IOL decentration measured by Pentacam and IOL decentration outcomes of OPD-Scan III.

Parameters	Mean ± SD (mm)	Mean SD	Repeat measurement (P)	ICC (95% Cl)
With reference to CTA (Pentacam)				
IOL decentration (x-axis)	-0.17 ± 0.12	0.06	0.92	0.82 (0.70–0.90)
IOL decentration (y-axis)	0.07 ± 0.11	0.06	0.43	0.76 (0.60–0.87)
IOL decentration (spatial distance)	0.24 ± 0.10	0.05	0.85	0.82 (0.70–0.90)
With reference to PC (Pentacam)				
IOL decentration (x-axis)	-0.20 ± 0.14	0.07	0.72	0.87 (0.78–0.93)
IOL decentration (y-axis)	0.09 ± 0.18	0.05	0.10	0.89 (0.81–0.94)
IOL decentration (spatial distance)	0.30 ± 0.13	0.04	0.70	0.77 (0.60–0.87)
With reference to CTA (OPD-Scan III)				
IOL decentration (x-axis)	-0.20 ± 0.22			
IOL decentration (y-axis)	0.28 ± 0.17			
IOL decentration (spatial distance)	0.25 ± 0.14			
With reference to PC (OPD-Scan III)				
IOL decentration (x-axis)	-0.32 ± 0.40			
IOL decentration (y-axis)	0.20 ± 0.35			
IOL decentration (spatial distance)	0.45 ± 0.27			

IOL, intraocular lens; SD, standard deviation; ICC, intraclass correlation coefficients; 95% CL, 95% confidence interval; CTA, corneal topographic axis; PC, pupil center.

**Fig 3:**
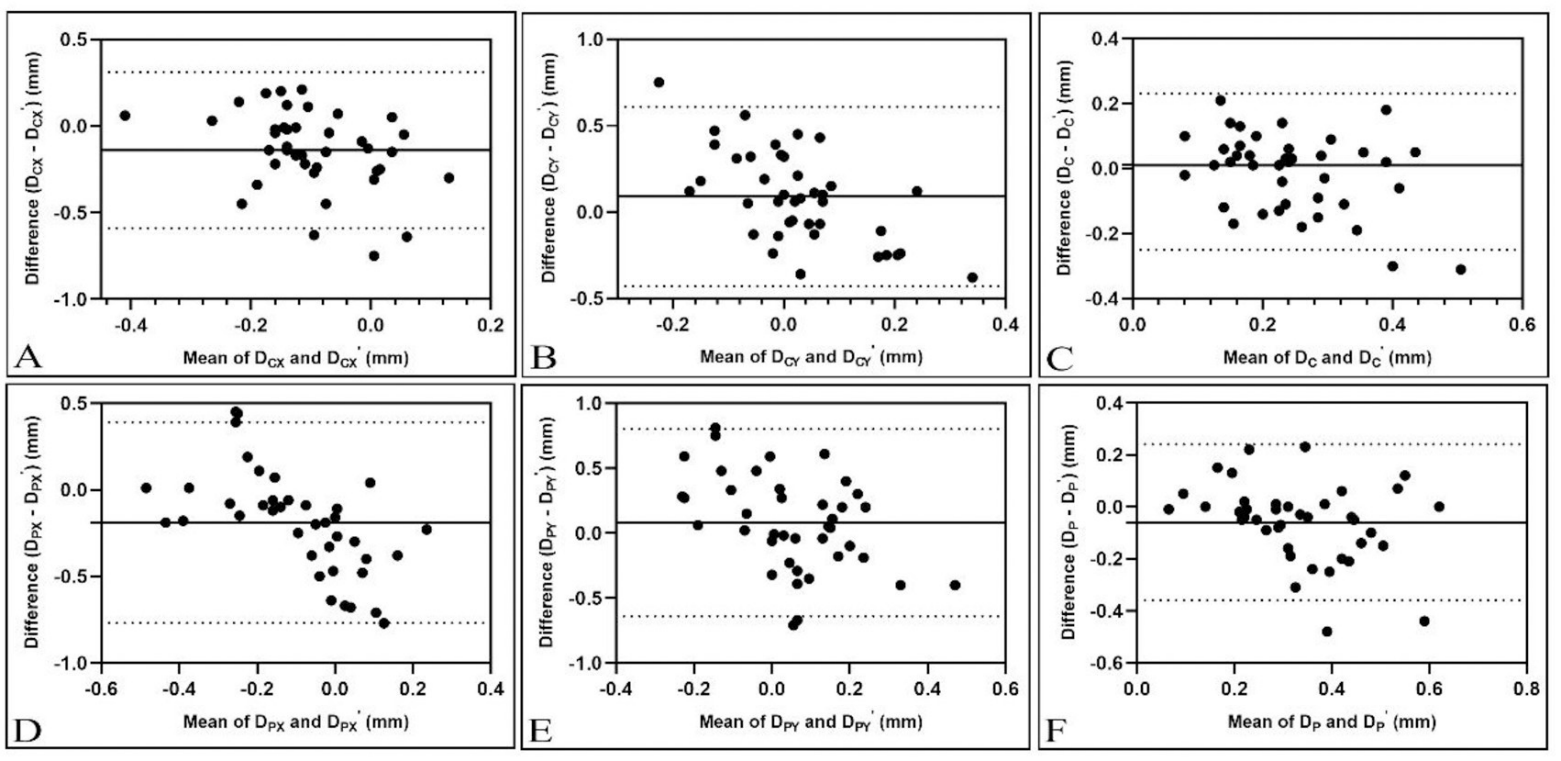
Bland-Altman plots for intraocular lens (IOL) decentration measurements for Pentacam and OPD-Scan III. The solid line indicates the mean difference (bias). The upper and lower lines represent the 95% limits of agreement. DCX, DCY and DC represent IOL decentration measured by Pentacam based on corneal topographic axis (CTA) in the x-axis, y-axis and spatial distance respectively. DPX, DPY and DP represent IOL decentration measured by Pentacam based on pupillary axis (PA) in the x-axis, y-axis and spatial distance respectively. DCX’, DCY’ and DC’ represent IOL decentration measured by OPD-Scan III based on CTA in the x-axis, y-axis and spatial distance respectively. DPX’, DPY’ and DP’ represent IOL decentration measured by OPD-Scan III based on PA in the x-axis, y-axis and spatial distance respectively.

The agreement of IOL decentration measurements between the Pentacam and OPD-Scan III was analyzed using Bland-Altman analysis (Fig 3). The 95% LoAs for all parameters were wide when comparing the three measurements, indicating poor agreement of IOL decentration between the two devices. Specifically, in the measurements of D_CX_, 20.0% of the measurement differences between the two devices exceeded 0.3 mm; in the measurements of D_CY_, 32.5% exceeded 0.3 mm; in the measurements of D_PX_, 40% exceeded 0.3 mm; in the measurements of D_PY_, 50% exceeded 0.3 mm; in the measurements of D_C_, 5% exceeded 0.3 mm; and in the measurements of D_P_, 7.5% exceeded 0.3 mm. It should be noted that the measurement differences of IOL decentration based on CTA were smaller than those based on the PA.

## Discussion

The evolution of cataract surgery into a refractive procedure has underscored the growing significance of accurate IOL decentration measurements for assessing optical outcomes [1, 4]. Decentration of the IOL can impair optical outcomes, such as higher-order aberrations (mainly coma) and visual quality metrics (e.g., Modulation Transfer Function and Point Spread Function) [4–7]. Therefore, accurate measurement is essential to avoid overestimating or underestimating the impact of IOL decentration on optical outcomes, especially for sophisticated optical IOLs [4–6]. Previous investigations have explored various axes, including the visual axis (VA), CTA and PA, as potential reference centers for such measurements [10, 17]. However, it is important to note that the visual axis represents a theoretical mathematical construct that lacks corresponding anatomical landmarks, thereby posing challenges in achieving clinical correlation with real eyes [25, 26]. Conversely, the CTA and PA axes, which possess well-defined anatomical characteristics and can be detected by commonly employed devices, including the Pentacam and the OPD-Scan III, have emerged as more frequently utilized parameters in clinical measurement practice [7, 10, 12, 13, 18]. Notably, to the best of our knowledge, no prior studies have specifically examined the repeatability of Pentacam-based IOL decentration measurements using CTA as a reference, nor the agreement between the Pentacam and the OPD-Scan III regarding IOL decentration measurements. Consequently, the primary objective of this study was to evaluate the repeatability of IOL decentration measurements obtained with the Pentacam. Additionally, we aimed to ascertain the level of agreement between the Pentacam and the OPD-Scan III devices in measuring IOL decentration, utilizing both CTA and PA as reference axes.

Our findings revealed that IOL decentration measurements centered on the CTA exhibited commendable repeatability, a notion supported by a previous study conducted by Fan Zhang et al., who proposed that the CTA may serve as the most visually pertinent reference axis [10]. Notably, when employing the Pentacam, a strong correlation was observed between visual acuity and CTA-based IOL decentration, contrasting with the use of PA as a reference [10]. Additionally, Arbelaez’s study reported improved postoperative visual acuity outcomes when aligning the ablation center with the CTA, further emphasizing the significance of clinical performance of CTA [27]. Coupled with the robust repeatability of IOL decentration measurements observed in our study, the CTA axis emerges as an optimal choice for IOL decentration assessment.

In parallel, favorable repeatability was also achieved when utilizing PA as a reference axis, a finding consistent with observations made by Castro et al. [16]. However, their study was limited in reliability as it relied solely on the pupil axis to evaluate IOL decentration, without considering the CTA. The primary drawback of employing PA as a reference lies in the variability of pupil size and center, influenced by factors such as light intensity, accommodation, pharmacological interventions, trauma, and surgical interventions, including mydriasis [17, 19, 20]. Notably, the strong repeatability observed in our study may largely be attributed to the standardized light levels and consistent mydriasis conditions maintained throughout the trial. Moreover, the measured PA center under experimental conditions deviates from its position in daily life, suggesting that IOL decentration centered on the PA may exhibit limited relevance to postoperative optical quality in patients’ everyday activities. Thus, PA may be considered as an adjunctive reference axis for the measurement of IOL decentration, particularly in situations characterized by aberrant corneal morphology, such as corneal scarring, ectasia, or previous corneal surgeries.

In our study, we observed a relatively wide 95% limit of agreement (LoA) between the Pentacam and OPD-Scan III. Notably, measurements based on the PA exhibited lower concordance than those based on the CTA for both devices. This discrepancy may be attributed to differences in pupil conditions resulting from variations in lighting levels between the two devices. Furthermore, the Pentacam displayed a tendency towards superotemporal IOL decentration and greater centralization. It is important to acknowledge that while the OPD-Scan III utilizes a 2-dimensional view for its measurements, the Pentacam employs a 3-dimensional approach, incorporating corrections for optical and geometric distortions during data processing [10]. We hypothesize that the corneal magnification evident in retroillumination images captured by the OPD-Scan III may significantly contribute to the observed differences. However, further research is necessary to validate this hypothesis.

In recent years, novel devices have been developed to assess postoperative IOL decentration [8, 28–30]. For instance, Ding et al. evaluated the repeatability of swept-source optical coherence tomography (SS-OCT) for IOL decentration and reported high ICCs when employing PA as the reference axis [28]. However, the limited availability of SS-OCT, particularly in regions such as China, hampers its widespread popularity as a tool for IOL decentration assessment.

Several limitations warrant mention. First, this study only compared two devices, and the feasibility of using alternative instruments interchangeably remains uncertain. Second, the absence of a gold standard comparison impedes a precise assessment of the accuracy of the two methods employed. Validation using a model eye might be advantageous in this regard. Thirdly, our study involved various types of IOLs. However, the four types of IOLs that we used were all 1-piece acrylic 360-degree square edge IOLs, which may have a negligible influence on IOL decentration measurements. Lastly, as the definition of IOL tilt differed between the two devices (physical tilt in the Pentacam and aberration parameters in the OPD-Scan III), the assessment of IOL tilt was not included in this study [15].

In conclusion, our study demonstrated the high repeatability of IOL decentration measurements obtained using the Pentacam, with both the CTA and PA serving as reliable reference axes. However, the poor agreement observed between the Pentacam and OPD-Scan III suggests that the interchangeable use of these two devices for IOL decentration measurement is not recommended.

## Supporting information

S1 Fig Definition for all parameters in retroillumination image using OPD-Scan III. The red box represents the distance and the orientation of intraocular lens center with respect to corneal topographic axis (CTA); The orange box represents pupil center with respect to CTA.
(DOCX)

S1 Data (XLSX)
