# Supplementary material for: Repeatability of Pentacam-derived intraocular lens decentration measurements and the level of agreement with OPD-Scan III: A prospective observational case series
Source: PLoS One. 2024 Mar 22;19(3):e0299064. doi: 10.1371/journal.pone.0299064 (PMC10959365; doi:10.1371/journal.pone.0299064)
Supplement: S1 Fig — The red box represents the distance and the orientation of intraocular lens center with respect to corneal topographic axis (CTA); The orange box represents pupil center with respect to CTA. (DOCX) [file pone.0299064.s001.docx]

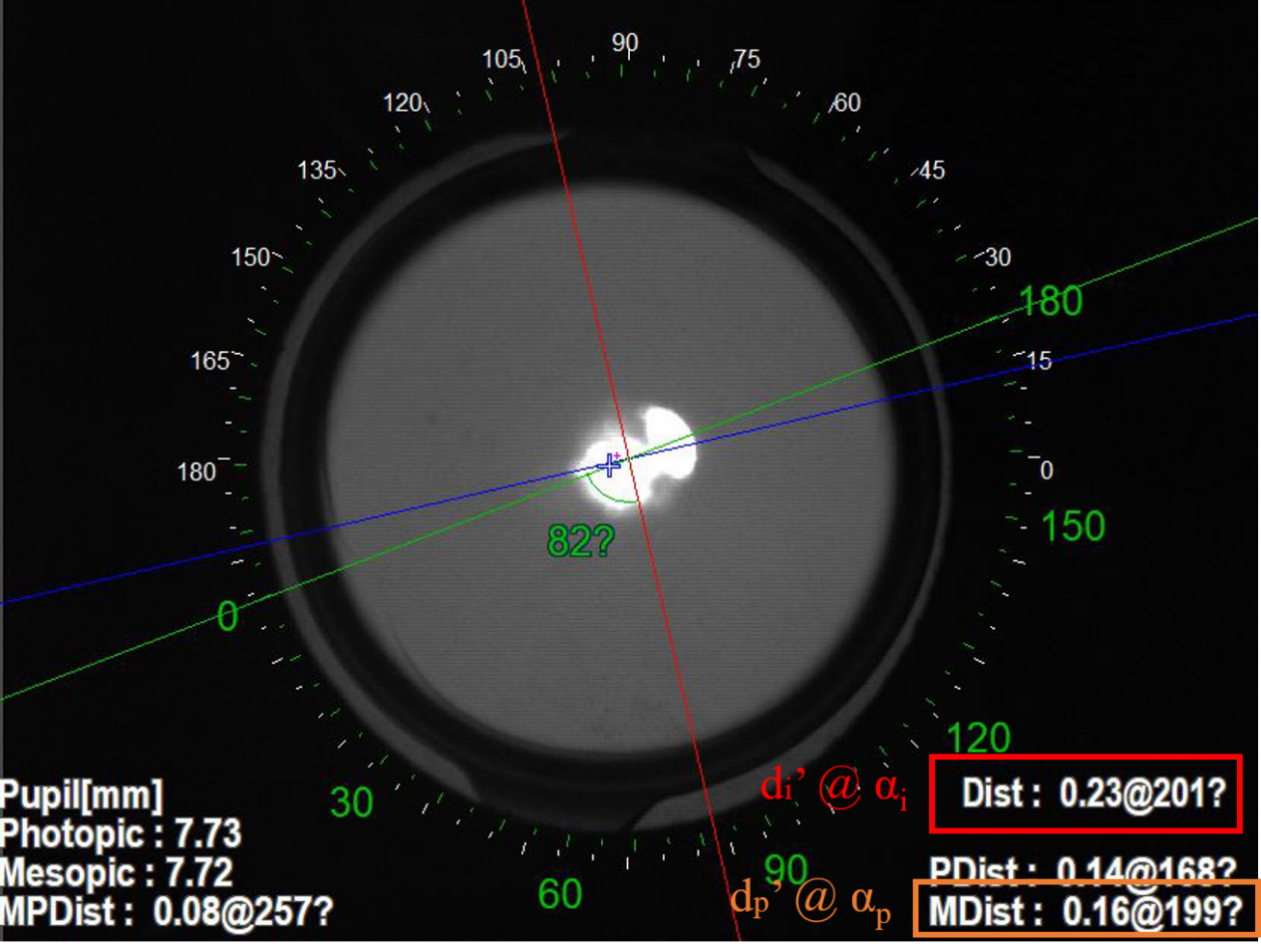


**S1 Figure. Definition for all parameters in retroillumination image using OPD-Scan III.** The red box represents the distance and the orientation of intraocular lens center with respect to corneal topographic axis (CTA); The orange box represents pupil center with respect to CTA.
